# Use of Benzodiazepines in Medical Students: A Comparative Analysis Between Medical and Other University Degrees

**DOI:** 10.3390/medsci13030164

**Published:** 2025-09-01

**Authors:** Paula Fernández de Frutos, Francisco Javier García-Sánchez, Natalia Mudarra-García, Fernando Roque-Rojas, Syed Ihtisham-Kakakhel, Davide Luordo-Tedesco

**Affiliations:** 1 Medical Department, Faculty of Medicine, University Complutense of Madrid, 28040 Madrid, Spain; paulfe20@ucm.es (P.F.d.F.); fernando.roque@salud.madrid.org (F.R.-R.); davidelu@ucm.es (D.L.-T.); 2 Emergency Room Service, Hospital Universitario Infanta Cristina, Instituto de Investigación Sanitaria Puerta de Hierro—Segovia Arana (IDIPHISA), 28981 Madrid, Spain; 3 Instituto Ramón y Cajal de Investigación Sanitaria (IRYCIS), 28034 Madrid, Spain; nmudarra@enf.ucm.es; 4 Nursing Department, Faculty of Nurse, Phisiotherapy and Podology, University Complutense of Madrid, 28040 Madrid, Spain; 5 Bacha Khan Medical Complex Swabi, Shahmansoor 23431, Pakistan; syed.ihtisham@bkmc.edu.pk

**Keywords:** benzodiazepines, psychotropic drugs, university students, medical students, academic stress, self-medication, dependence, mental health

## Abstract

Background: The use of benzodiazepines among university students has been scarcely investigated. This situation raises particular concerns in medical students, due to their exposure to stressful situations and, especially, their familiarity with psychotropic drugs. Material and methods: A descriptive cross-sectional observational study was conducted using an anonymous online survey disseminated among universities in the Community of Madrid during April 2024. Results: 25.07% of students stated they had used benzodiazepines at least once, especially from the third academic year onwards. The prevalence was higher among medical students (32.34%). Use was mainly occasional, although 20.21% reported daily use. Among the reasons for use, managing academic stress reached 45.74%. Up to 15.96% of respondents reported a feeling of dependence, and 32.26% noticed concentration difficulties as a side effect of benzodiazepine use. Conclusions: Benzodiazepine use is a relevant phenomenon among university students, with particular incidence in medical degrees. Its onset usually coincides with advanced stages of the degree, which underscores the need for preventive interventions tailored to the academic environment and for the rational use of psychotropic drugs in young populations.

## 1. Introduction

Benzodiazepines are central nervous system depressants commonly prescribed for anxiety, insomnia, and muscle spasms. Their use among young populations raises concerns due to dependence and cognitive impairment risks, especially when used without medical supervision. In Spain, benzodiazepines require a medical prescription, although informal access routes such as leftover prescriptions exist (non-medical access may occur through leftover prescriptions, family members, or informal sharing, as documented in other European studies).

The consumption of psychoactive substances among university students must be understood within the broader context of the high prevalence of mental health disorders in this population, particularly anxiety, depression, and substance use disorders [1]. Benzodiazepines—central nervous system depressants with anxiolytic, hypnotic, and muscle relaxant properties—are part of this phenomenon.

In recent years, several studies have documented the non-prescribed use of psychotropic medications as a strategy employed by students to cope with academic stress, insomnia, and the pressure to maintain high academic performance [2]. This pattern of consumption, often perceived as a form of self-medication or as a seemingly harmless aid, is facilitated by easy access to these drugs and a low perception of risk, which increases the potential for abuse and dependence [3].

Despite growing interest in this issue, the scientific literature still presents important gaps. In particular, there are few studies that compare benzodiazepine use between medical students and students from other university degrees. This distinction is especially relevant, as medical students tend to experience higher levels of anxiety, as well as greater familiarity with the use and prescription of psychotropic drugs [4]. Moreover, many existing studies rely on small or non-representative samples, which limits the identification of risk profiles associated with specific academic disciplines. Additionally, it is uncommon for these studies to examine in detail aspects such as personal motivations, access routes, or the subjective perception of dependence.

Given these limitations, this study aims to compare the prevalence of benzodiazepine use between medical students and students from other university degrees in the Community of Madrid. Secondary objectives include estimating the overall use of psychoactive substances for managing academic stress, identifying the most frequently used substances for this purpose, describing the main access routes to these substances, analyzing the frequency and motivations for benzodiazepine use, and exploring its association with cognitive symptoms such as difficulties in concentration and the subjective perception of dependence.

## 2. Materials and Methods

### 2.1. Study Design

This was a descriptive, cross-sectional observational study conducted within the Teaching Unit of the 12 de Octubre–Infanta Cristina University Hospitals, Madrid, Spain.

This study was conducted in accordance with the ethical principles of the Declaration of Helsinki. The Ethics Committee of Puerta de Hierro University Hospital approved both the study protocol and the full questionnaire content, including items regarding non-medical and illicit drug use (Reference 54/24 ACT 06/2024). Participation in the study was entirely voluntary, and informed consent was obtained digitally before completing the survey. The questionnaire was anonymous and did not collect any personal identifiers. Respondents were informed about the study’s purpose, data confidentiality, and their right to withdraw at any time without justification. No sensitive personal data were collected, and no clinical intervention or diagnostic assessment was performed. Therefore, no written informed consent was required according to applicable local regulations for research involving anonymized survey data in university settings.

Data collection was carried out between 1 April and 30 April 2024, through an anonymous online survey developed using Microsoft Forms™. The questionnaire consisted of 19 questions (17 single-answer and 2 multiple-answer items) and was disseminated via social media platforms and university forums, aiming to reach a broad and diverse sample. Microsoft Forms™ was configured to guarantee full anonymity by disabling IP tracking, login requirement, and email collection. No identifying data were stored. This platform was accepted by the Ethics Committee for its data security features.

### 2.2. Study Population

The target population was university students enrolled in institutions located in the Community of Madrid. The only inclusion criterion was being enrolled, at the time of the survey, in a Bachelor’s degree program offered by a public or private university, the Distance University of Madrid (UDIMA), or the Madrid and Madrid-South centers of the National Distance Education University (UNED). Students under the age of 18 were excluded.

### 2.3. Sample Size

To determine the appropriate sample size, the proportion of medical students within the total student population of the Community of Madrid was estimated. According to official data, there were 358,881 enrolled university students during the 2023–2024 academic year, with approximately 9037 of them enrolled in medical programs [5,6]. This figure was extrapolated from the proportion observed in the previous academic year (2.52%).

The sample size calculation was based on previous studies estimating a 4.50% prevalence of non-medical benzodiazepine use in the general university population, compared to 0.90% among medical students [7]. Assuming a 95% confidence level, 80% statistical power, and the need to compare two proportions, a minimum sample of 317 students was calculated. To account for potential exclusions or invalid responses, a 15% margin was added, establishing a final target of 373 participants.

Although no formal dropouts occurred, several responses were excluded at the beginning of the analysis for not meeting the inclusion criteria. Specifically, questionnaires completed by individuals not enrolled in university education—mostly vocational training students—were excluded prior to statistical processing. As a result, the final valid sample consisted of 373 participants, meeting the pre-established target.

### 2.4. Studied Variables

The survey included both sociodemographic and substance use-related variables. Sociodemographic variables comprised age, gender, and degree program (medicine or other health-related and non-health-related degrees). Variables related to benzodiazepine use included awareness of the drug, history of use, frequency and context of consumption (e.g., academic stress, sleep difficulties, clinical diagnosis), route of acquisition (prescribed or non-prescribed), and perception of side effects. For participants who reported benzodiazepine use, additional questions explored dosage regularity, timing of initiation, and subjective experience of dependence or control. The questionnaire also inquired about the use of other psychoactive substances, including alcohol, cannabis, and antidepressants, for academic stress management.

### 2.5. Intervention

The intervention consisted of the dissemination of an anonymous, self-administered online questionnaire developed ad hoc for the study. The survey was distributed through institutional university mailing lists and academic forums in the Community of Madrid during the academic year 2022–2023. Participation was voluntary and students were informed of the study’s objectives, the estimated time to complete the survey, and the anonymity of their responses. No incentives were provided. The questionnaire was designed to collect detailed data regarding sociodemographic characteristics, academic context, benzodiazepine and other psychoactive substance use, and perceived effects. No clinical or pharmacological intervention was performed, and the study was observational in nature. The full survey instrument (Spanish original with English translation) is provided in Supplementary File S1.

### 2.6. Statistical Analysis

Statistical analysis was performed using JAMOVI^®^ software (version 2.3.28). Descriptive techniques were applied to characterize the sample and the main study variables. The chi-squared test was used to identify statistically significant differences in benzodiazepine use between medical and non-medical students, as well as by gender.

A large language model (ChatGPT, version GPT-4o, OpenAI, August 2025 release, San Francisco, CA, USA) was used to support English language refinement, improve sentence structure, and assist in organizing the visual presentation of figures and tables. All outputs were reviewed and verified by the authors.

## 3. Results

### 3.1. Sample Characteristics

The final sample consisted of 373 university students. The mean age was 21.64 years (SD = 1.93), and participants were distributed across four age groups: 18–20, 21–23, 24–26, and over 26 years. The most common age range was 21–23 years. Regarding gender, 69.10% identified as female, 30.40% as male, and 0.50% preferred not to specify (Table 1).

**Figure 1:**
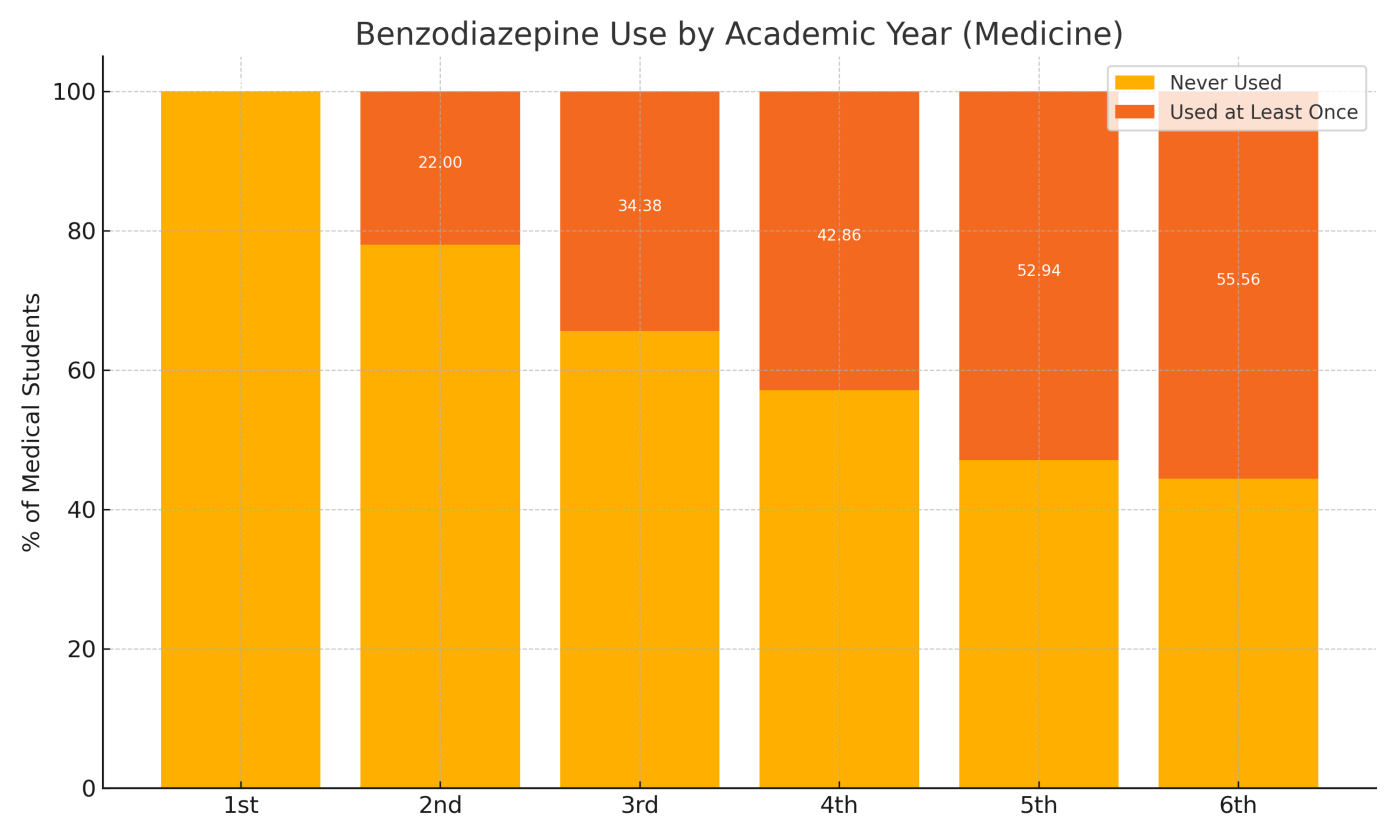
Benzodiazepine use by academic year (Medicine students).

With respect to academic year, 5.33% of students were in their first year, 13.87% in second, 18.13% in third, 36.00% in fourth, 18.40% in fifth, and 7.20% in sixth year. A small proportion (1.07%) did not respond to this item. The most frequent profile was a female student aged 21–23 years enrolled in the fourth year of her degree (Figure 1). Overall, 53.60% of the sample were enrolled in a medical degree (Table 2).

### 3.2. Use of Psychoactive Substances

**Figure 2:**
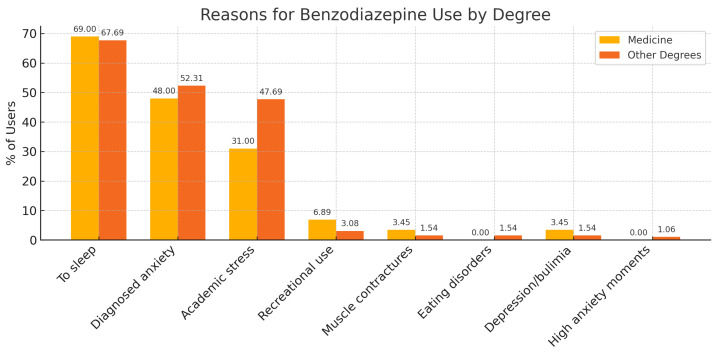
Reasons for benzodiazepine use among university students.

Among the respondents, 78.93% were aware of what benzodiazepines are, and 32.27% had used benzodiazepines or other psychoactive substances to manage stress related to their studies (Table 3). Among these, benzodiazepines were the most commonly used substance, followed by alcohol, antidepressants, and cannabis (Table 4 and Figure 2).

### 3.3. Frequency and Patterns of Benzodiazepine Use

A total of 25.07% of students reported having used benzodiazepines at least once. This percentage was higher among medical students (32.34%) compared to students from other degrees (16.67%) (Table 5).

**Figure 3:**
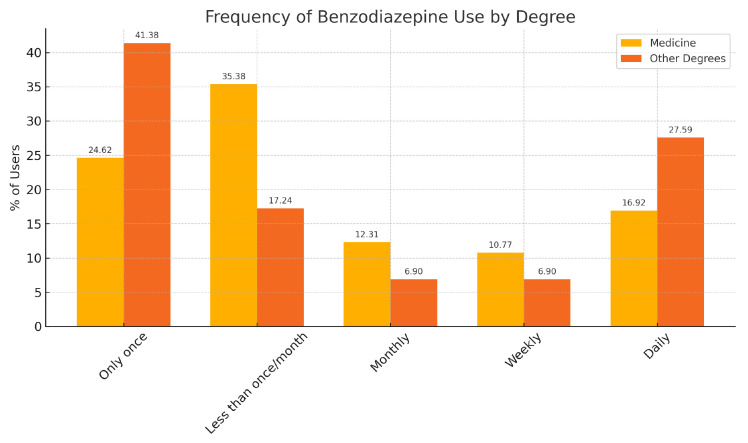
Frequency of benzodiazepine use by degree.

Among those who had used benzodiazepines, 29.79% reported using them only once, and another 29.79% used them less than once per month. Daily use was reported by 20.21% of these respondents. Weekly and monthly use were reported by 9.57% and 10.64%, respectively (Figure 3).

When comparing medical students with students from other degrees, the most common pattern among medical students was using benzodiazepines less than once a month, whereas among non-medical students, the most common pattern was having used them only once. Interestingly, daily use was slightly more common in non-medical degrees than in medicine (Table 6).

### 3.4. Adverse Effects

**Figure 4:**
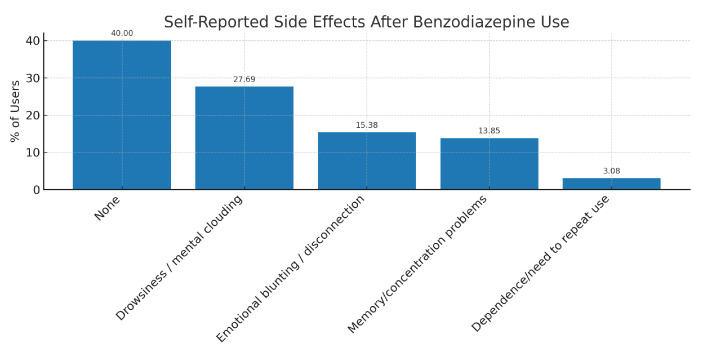
Self-reported side effects after benzodiazepine use.

Among students who had used benzodiazepines, 60.00% reported experiencing at least one adverse effect. The most commonly cited symptoms were drowsiness or mental clouding, emotional blunting, and difficulties with memory or concentration. A full breakdown of these self-reported side effects is presented in Table 7 and illustrated in Figure 4.

## 4. Discussion

Discussion This study documents a high prevalence of benzodiazepine (BZD) use among university students in the Community of Madrid and shows that medical students report higher lifetime use than their peers from other degrees (32.34% vs. 16.67%). In the overall sample, one in four students (25.07%) had used BZDs at least once, and 60% of users reported at least one adverse effect, most commonly drowsiness/mental clouding, emotional blunting, and concentration or memory difficulties. Although the most frequent pattern was occasional use, a non-negligible subgroup reported daily intake (20.21% among users), underscoring potential risks of dependence and cognitive side effects. These findings, while limited to a regional sample and a cross-sectional design, add empirical data to a sparsely explored setting in Spain and help refine targets for prevention within academic environments.

A first contribution of this work is the explicit comparison between medical and non-medical students. Higher BZD use among medical students is consistent with prior evidence of elevated stress, sleep disruption, and greater familiarity with pharmacological agents in medical education [8,9]. Several mechanisms may explain this gradient. First, academic load and evaluation intensity typically increase across the degree, particularly during clinical years, potentially reinforcing pharmacological coping for insomnia and exam-related anxiety. Second, clinical exposure may normalize prescribed BZDs as legitimate short-term tools, shaping attitudes via informal learning processes within health professions education; this aligns with research on student perceptions and motives for non-medical prescription drug use [10]. Third, proximity to healthcare settings can facilitate awareness of drug effects, dosages, and access pathways, even when formal prescribing is not intended for the student. Notably, the most cited reasons for use in our sample were sleep facilitation and management of academic stress—patterns consistent with psychotropic self-medication in young adults [11].

A second relevant finding is the progressive increase in BZD experience with advancing academic year among medical students—reaching more than half of students in the final year—suggesting a dose–response–like pattern with cumulative academic pressure and clinical exposure. While our design cannot establish causality, the monotonic trend supports hypotheses that both perceived stress and perceived acceptability of pharmacological coping rise over time. In non-medical programs, where clinical exposure is absent and access routes may be more informal, the pattern may diverge; in our data, non-medical students more often reported having tried BZDs only once, whereas medical students more commonly reported sporadic (less than monthly) repeated use. These nuances argue for tailoring preventive messages to program-specific contexts: in medical programs, emphasis on professional identity formation, pharmacovigilance, and non-pharmacological coping; in other degrees, emphasis on risk perception and safe access.

The adverse-effects profile reported by students aligns with established BZD pharmacodynamics and prior syntheses of cognitive impact with prolonged or unsupervised use [12,13]. That 60% of users perceived at least one side effect (with cognitive complaints in 14% of users) is noteworthy for populations whose academic performance depends heavily on sustained attention and working memory. At the same time, the relatively low self-reported perception of dependence suggests risk underestimation—possibly due to familiarity bias, normalization within peer groups, or the fact that a share of use was occasional and linked to exam periods. Education about short-term benefits versus medium-term risks (tolerance, rebound insomnia/anxiety, cognitive slowing) may help recalibrate risk–benefit appraisals among students and prescribers.

Our results also intersect with the broader issue of non-medical use and polydrug behaviors in youth. Although BZDs were the most frequently reported substance for academic stress among users in our survey, alcohol, antidepressants, and cannabis were also common. Even without detailed temporal sequencing, the co-occurrence of these substances raises safety concerns: combined CNS depressants (e.g., BZDs and alcohol) increase risks of accidents, impaired academic functioning, and misuse [3,13,14]. Future studies should characterize polydrug patterns more precisely (timing, contexts such as nightlife vs. exam periods, motives) to inform targeted, situational prevention (e.g., pre-exam sleep hygiene vs. weekend harm-reduction messages).

Gender deserves attention. The sample was predominantly female (69%), and gender-stratified descriptive analyses (added in the revised Results) suggested patterns consistent with prior literature reporting higher anxiety, sleep difficulties, and psychotropic use among women [8]. However, given the sample composition and the descriptive nature of our analyses, effect sizes should be interpreted cautiously. Future work should balance samples by gender, incorporate validated measures of anxiety and insomnia, and test interaction effects between gender and academic year or degree type.

From an ethics and professional-development perspective, our findings invite reflection on how medical training addresses stress, sleep, and pharmacological literacy. If BZDs become normalized as a quick solution to academic demands, the habit may carry into residency and practice, with implications for clinician well-being, patient safety (fatigue, attentional lapses), and role-modeling to junior students. Curricular strategies could therefore combine

- Structured psychoeducation about BZDs (indications, short-term use limits, tapering, dependence risks);
- Skills for non-pharmacological management (CBT-I principles, stimulus control, sleep hygiene, mindfulness-based stress reduction);
- Reflective exercises on professional identity and ethical prescribing.

At the institutional level, easily accessible campus mental-health services, screening for insomnia/anxiety during high-risk periods, and campaigns to discourage sharing leftover prescription medications could help reduce non-prescribed access.

Methodologically, this study’s strengths include its adequate sample size (matching the a priori calculation), the inclusion of both medical and non-medical students, and the capture of both motives and self-perceived side effects. Nevertheless, key limitations temper inference and guide future improvements. The online self-administered format, while appropriate for sensitive topics, likely introduced self-selection and social-desirability biases; all measures were self-reported and lacked clinical validation and the questionnaire had not been pilot-tested prior to dissemination. Moreover, the study is geographically limited to Madrid, and several potentially relevant behaviors (e.g., detailed polydrug patterns or leisure-context consumption) were not fully characterized. These constraints, now explicitly acknowledged, justify a cautious interpretation and motivate more robust designs.

Implications for policy and practice follow naturally. In Spain, BZDs require medical prescription; our data suggest that a portion of student use occurs through informal access (e.g., leftover family prescriptions or peer sharing). Interventions could therefore combine stronger messages on safe storage and disposal of prescription medications, reminders against sharing prescriptions, and prescriber guidance to limit quantities and plan discontinuation. Within universities, establishing or reinforcing stepped-care models (low-intensity digital CBT for insomnia and anxiety; brief counseling; referral pathways) may reduce reliance on pharmacological coping. For medical faculties, embedding micro-modules on deprescribing, sleep medicine, and healthy rota planning during clinical years can align personal health with professional standards.

Finally, our results reinforce priorities for future research. Multi-site, larger samples would improve generalizability beyond a single region; longitudinal designs are needed to map trajectories across academic years and exam cycles; validated, pilot-tested instruments should be used to enhance measurement quality; and mixed-methods approaches (focus groups, interviews) could illuminate motivations, social norms, and perceived barriers to non-pharmacological strategies. Given the signal for daily use in a subset of students and the potential for polydrug combinations, analytical attention to clustering of behaviors and to periods of heightened risk (e.g., finals) is warranted.

In summary, within a Madrid university sample, BZD use is common—especially among medical students and in advanced academic years—and is frequently motivated by sleep and stress management. Reported adverse effects are substantial, and non-prescribed access is present. While these findings are not generalizable to all student populations, they indicate clear opportunities for targeted prevention in universities and for curricular initiatives in medical education to promote safer, evidence-based coping strategies.

## 5. Conclusions

This study reveals a high prevalence of benzodiazepine use among university students in the Community of Madrid, particularly in medical students and in advanced academic years. Although most reported occasional use, daily intake and non-prescribed access were also present, raising concerns about dependence and cognitive side effects. These findings highlight the importance of preventive strategies tailored to university populations, with special attention to medical education. The results should be interpreted within the context of a single regional sample and a cross-sectional design, and therefore cannot be generalized to all university students. Future studies should incorporate broader populations, longitudinal designs, and pilot-tested instruments to improve validity and generalizability.

## 6. Limitations

This study has several limitations. First, the use of a self-administered online questionnaire may have introduced self-selection bias, as students with prior experience or interest in psychoactive substances could have been more likely to respond. Second, all data were self-reported, which increases the risk of recall bias and social desirability bias, particularly given the sensitive nature of substance use. Third, the questionnaire was not pilot-tested before dissemination, which may have limited the ability to identify potential issues with clarity or interpretation of questions. Fourth, the study was geographically limited to the Community of Madrid, which restricts the generalizability of the results. Fifth, although the survey included a wide range of substances, it did not explore in depth patterns of polydrug use, recreational contexts, or leisure-related consumption, which should be addressed in future studies. Finally, no clinical validation of psychiatric diagnoses or substance dependence criteria was performed, as the study relied exclusively on subjective responses. Despite these limitations, the study provides valuable exploratory insights into benzodiazepine use among university students in Spain.

## 7. Future Directions

Future research on benzodiazepine use among university students should incorporate several methodological improvements. First, survey instruments should be pilot-tested before dissemination to ensure clarity and reliability. Second, larger and more diverse samples across multiple universities and regions would increase the generalizability of findings. Third, longitudinal designs are needed to track patterns of use over time and to establish temporal associations with academic stress and progression through university studies. Fourth, more detailed assessment of polydrug use, recreational contexts, and leisure-related consumption is warranted, given their potential influence on risk behaviors. Fifth, future studies should explore gender differences and discipline-specific factors more thoroughly, as these may reveal distinct vulnerability profiles. Finally, integrating qualitative approaches, such as focus groups or interviews, could provide richer insights into motivations, perceptions of risk, and coping strategies, complementing the quantitative evidence and informing the development of tailored preventive interventions within academic environments.

## Figures and Tables

**Table 1:** Summary of key descriptive variables of the study population (n = 373).

Variable	Category/Statistic	Value
Age (mean ± SD)	Years	21.64 ± 1.93
Gender	Female	69.10%
	Male	30.40%
	Non-disclosed	0.50%
Degree program	Medicine	53.60%
	Other degrees	46.40%
Benzodiazepine awareness	Knows what they are	78.93%
Use of substances for stress	Any substance	32.27%
	Benzodiazepines	23.47%
	Alcohol	17.33%
Benzodiazepine use (ever)	Yes	25.07%
	Medicine students	32.34%
	Other students	16.67%
Subjective side effects reported	None	40.00%
	Drowsiness, etc.	60.00% (combined)

**Table 2:** Prevalence of benzodiazepine use by academic year among medical students.

Academic Year	Never Used (%)	Used at Least Once (%)
First Year	100.00	0.00
Second Year	78.00	22.00
Third Year	65.63	34.38
Fourth Year	57.14	42.86
Fifth Year	47.06	52.94
Sixth Year	44.44	55.56

**Table 3:** Main reasons for benzodiazepine use among university students by degree.

Reason for Use	Medicine (%)	Other Degrees (%)
To facilitate sleep	69.00	67.69
To treat medically diagnosed anxiety	48.00	52.31
To manage academic stress	31.00	47.69
Recreational use	6.89	3.08
Muscle contractures	3.45	1.54
Eating disorders (ED)	0.00	1.54
Depression and bulimia	3.45	1.54
High anxiety in specific moments	0.00	1.06

**Table 4:** Types of psychoactive substances used by university students.

Substance	Absolute Frequency	% of Total Respondents	% Among Users for Academic Stress
Benzodiazepines	88	23.47%	72.73%
Alcohol	65	17.33%	53.72%
Antidepressants	47	12.53%	38.84%
Cannabis	37	9.87%	30.58%
MDMA	9	2.40%	7.44%
Antipsychotics	5	1.33%	4.13%
Cocaine	3	0.80%	2.48%
Illegal amphetamines	3	0.80%	2.48%
Hallucinogenic mushrooms	3	0.80%	2.48%
Methylphenidate	3	0.80%	2.48%
Ketamine	2	0.53%	1.65%
Methamphetamine	2	0.53%	1.65%
Amphetamines	2	0.53%	1.65%
None of the above	3	0.80%	2.48%

**Table 5:** Prevalence of benzodiazepine use among university students by degree.

Degree Program	Never Used (%)	Used at Least Once (%)
Medicine	67.66	32.34
Other Degrees	83.33	16.67

**Table 6:** Frequency of benzodiazepine use among students who reported any use.

Frequency of Use	Medicine (%)	Other Degrees (%)
Only once	24.62	41.38
Less than once/month	35.38	17.24
Monthly	12.31	6.90
Weekly	10.77	6.90
Daily	16.92	27.59

**Table 7:** Self-reported side effects after benzodiazepine use among university students.

Reported Side Effect	Frequency (%)
None	40.00
Drowsiness/mental clouding	27.69
Emotional blunting/disconnection	15.38
Memory or concentration problems	13.85
Dependence or feeling the need to repeat use	3.08

## Data Availability

The original contributions presented in this study are included in the article/Supplementary Material. Further inquiries can be directed to the corresponding author.

## Supplementary Materials

The following supporting information can be downloaded at https://www.mdpi.com/article/10.3390/medsci13030164/s1, Supplementary File S1: Survey Instrument (Spanish original and English translation); Supplementary File S2: Original Microsoft Forms™ PDF export of the questionnaire.

## Abbreviations

The following abbreviations are used in this manuscript:

UDIMA	Distance University of Madrid
UNED	National Distance Education University
