# Supplementary material for: Use of Benzodiazepines in Medical Students: A Comparative Analysis Between Medical and Other University Degrees
Source: Med Sci (Basel). 2025 Sep 1;13(3):164. doi: 10.3390/medsci13030164 (PMC12452709; doi:10.3390/medsci13030164)
Supplement: Supplementary file 1 [file medsci-13-00164-s001.zip › Supplementary File S1. Survey Instrument.pdf]

Supplementary Material S1: Survey Instrument (Full Version)

Study: Use of Benzodiazepines in Medical Students: A Comparative Analysis Between Medical and Other University Degrees

Ethics approval: Puerta de Hierro University Hospital Ethics Committee (54/24 ACT 06/2024)

Original instrument administered in Spanish via Microsoft Forms™. This annex reproduces the full consent text and questionnaire items, with English translations for transparency.

A) Consentimiento informado — Original (Español)

CONSENTIMIENTO INFORMADO

Título del Estudio: Consumo de benzodiacepinas en estudiantes de medicina: un análisis comparativo entre estudiantes de medicina y otros grados universitarios.

Investigadores Principales: Paula Fernández de Frutos, estudiante de medicina, Universidad Complutense de Madrid; Davide Luordo Tedesco, Profesor asociado a ciencias de la salud, Departamento de Medicina, Universidad Complutense de Madrid.

Descripción del Estudio: Le invitamos cordialmente a participar en una encuesta diseñada para investigar el uso de sustancias psicoactivas, con un enfoque particular en las benzodiacepinas, entre estudiantes universitarios. Las benzodiacepinas son medicamentos psicotrópicos sintéticos, usados en medicina para tratar problemas de ansiedad como el trastorno de ansiedad, el estrés o el insomnio. Algunas de las más comunes son diazepam (Valium), alprazolam (Trankimazin) y clonazepam (Rivotril). El objetivo principal es analizar la prevalencia de consumo de dichas sustancias entre estudiantes de medicina y compararlo con el de estudiantes de otras disciplinas universitarias. Además, nos interesa determinar las vías de acceso, explorar la relación entre su consumo y el impacto en la atención, concentración y memoria, e identificar el uso sin prescripción médica.

Procedimiento: Si acepta participar, se le pedirá que complete una encuesta en línea sobre su uso de benzodiacepinas y otras sustancias psicotrópicas. Estimar el tiempo de cumplimentación en 10–15 minutos.

Voluntariedad y Confidencialidad: Su participación es completamente voluntaria. Puede retirarse en cualquier momento sin necesidad de dar una razón y sin consecuencias. Todas las respuestas serán tratadas con la máxima confidencialidad y anonimato. Los datos se utilizarán únicamente con fines de investigación y no se compartirán con terceros fuera del equipo investigador.

Beneficios y Riesgos: Su participación ayudará a comprender mejor el uso de sustancias

psicoactivas en el contexto universitario y podrá contribuir a políticas de salud pública mejor informadas. No se anticipan riesgos significativos.

Consentimiento: Al comenzar la encuesta, usted otorga su consentimiento informado para participar en este estudio. Para cualquier duda acerca del estudio o de sus derechos como participante, por favor contacte con los investigadores.

Contacto: Davide Luordo (davidelu@ucm.es; 692903919).

B) Informed Consent — English Translation

Informed Consent

Study Title: Benzodiazepine use in medical students: a comparative analysis between medical and other university degrees.

Principal Investigators: Paula Fernández de Frutos (medical student, Complutense University of Madrid); Davide Luordo Tedesco (Assistant Professor in Health Sciences, Department of Medicine, Complutense University of Madrid).

Study Description: You are invited to participate in an online survey investigating psychoactive substance use among university students, with a particular focus on benzodiazepines. Benzodiazepines are synthetic psychotropic medicines used to treat anxiety, stress and insomnia. Common examples include diazepam (Valium), alprazolam (Trankimazin) and clonazepam (Rivotril). The primary aim is to compare prevalence between medical students and those from other disciplines. Secondary aims include access routes, associations with attention, concentration and memory, and identification of non-prescribed use.

Procedure: If you agree to participate, you will complete an online questionnaire. Estimated completion time is 10–15 minutes.

Voluntariness and Confidentiality: Participation is entirely voluntary, and you may withdraw at any time without consequences. All responses are anonymous and confidential. Data will be used solely for research purposes and not shared outside the research team.

Benefits and Risks: Your participation will help improve understanding of psychoactive substance use in university settings and may inform public health policies. No significant risks are anticipated.

Consent: By starting the survey, you provide informed consent to participate. For questions about the study or your rights as a participant, please contact the investigators.

Contact: Davide Luordo (davidelu@ucm.es; +34 692903919).

C) Questionnaire Items (Spanish original and English translation)

Q1. He leído el consentimiento informado y acepto participar en la encuesta. *

- Sí

- No

English: I have read the informed consent and agree to participate in the survey.

- Yes

- No

Q2. EDAD *

- 18–20 años

- 21–23 años

- 24–26 años

- > 26 años

English: AGE

- 18–20 years

- 21–23 years

- 24–26 years

- > 26 years

Q3. SEXO *

- Hombre

- Mujer

- Prefiero no decirlo

English: GENDER

- Male

- Female

- Prefer not to say

Q4. UNIVERSIDAD DE ORIGEN *

- U. Alcalá de Henares (UAH)

- U. Autónoma de Madrid (UAM)

- U. Carlos III de Madrid (UC3M)

- U. Complutense de Madrid (UCM)

- U. Politécnica de Madrid (UPM)

- U. Rey Juan Carlos (URJC)

- U. Alfonso X el Sabio (UAX)

- U. Antonio Nebrija (Nebrija)

- U. Camilo José Cela (UCJC)

- U. CEU San Pablo (CEU)

- U. Europea de Madrid (UEM)

- U. Francisco de Vitoria (UFV)

- U. Pontificia de Comillas (UPComillas)

- U. a distancia de Madrid (UDIMA)

- ESIC University

- Otra

English: UNIVERSITY OF ORIGIN

- UAH

- UAM

- UC3M

- UCM

- UPM

- URJC

- UAX

- Nebrija

- UCJC

- CEU

- UEM

- UFV

- UPComillas

- UDIMA

- ESIC University

- Other

Q5. GRADO AL QUE PERTENECES *

- ADE

- Derecho

- Ingeniería Informática

- Psicología

- Enfermería

- Educación primaria

- Arquitectura

- Medicina

- Ingeniería industrial

- Comunicación audiovisual

- Fisioterapia

- Biología

- Química

- Educación infantil

- Ingeniería civil

- Farmacia

- Filología hispánica

- Ciencias ambientales

- Física

- Otro

English: DEGREE PROGRAM

- Business Administration

- Law

- Computer Engineering

- Psychology

- Nursing

- Primary Education

- Architecture

- Medicine

- Industrial Engineering

- Audiovisual Communication

- Physiotherapy

- Biology

- Chemistry

- Early Childhood Education

- Civil Engineering

- Pharmacy

- Hispanic Philology

- Environmental Sciences

- Physics

- Other

Q6. DURACIÓN DEL GRADO *

- 4 cursos

- 5 cursos

- 6 cursos

- Otra

English: PROGRAM DURATION

- 4 years

- 5 years

- 6 years

- Other

Q7. CURSO ACADÉMICO (marca el curso con más asignaturas matriculadas) *

- 1º

- 2º

- 3º

- 4º

- 5º

- 6º

English: ACADEMIC YEAR (mark the year in which you are enrolled in the most credits)

- Year 1

- Year 2

- Year 3

- Year 4

- Year 5

- Year 6

Q8. ¿Sabes lo que son las benzodiacepinas? *

- Sí

- No

English: Do you know what benzodiazepines are?

- Yes

- No

Q9. ¿Has tomado alguna vez alguna sustancia psicoactiva (alcohol, drogas, fármacos como las benzodiazepinas) para combatir el estrés de tus estudios? *

- Sí

- No

English: Have you ever taken any psychoactive substance (alcohol, drugs, medicines such as benzodiazepines) to cope with study-related stress?

- Yes

- No

Q10. ¿Cuál de las siguientes has usado al menos una vez? (selección múltiple) *

- Alcohol

- Cannabis

- Cocaína

- MDMA

- Anfetaminas ilegales

- LSD

- Hongos alucinógenos

- Ketamina

- Opiáceos

- Metanfetamina

- Metilfenidato

- Modafinilo

- Benzodiazepinas

- Antidepresivos

- Antipsicóticos

- Ninguna de las anteriores

English: Which of the following have you used at least once? (multiple selection)

- Alcohol

- Cannabis

- Cocaine

- MDMA

- Illegal amphetamines

- LSD

- Hallucinogenic mushrooms

- Ketamine

- Opioids

- Methamphetamine

- Methylphenidate

- Modafinil

- Benzodiazepines

- Antidepressants

- Antipsychotics

- None of the above

Q11. ¿Cómo accediste a las sustancias seleccionadas (sin considerar las drogas de abuso)? *

- Prescripción médica

- A través de familiar/amigo

- Solo he consumido drogas consideradas de abuso

- Otra

English: How did you access the selected substances (excluding drugs of abuse)?

- Medical prescription

- Via family/friend

- I have only used drugs considered as abuse/illicit drugs

- Other

Q12. ¿Has consumido alguna vez benzodiazepinas? *

- Sí

- No

English: Have you ever used benzodiazepines?

- Yes

- No

Q13. ¿Con qué frecuencia consumes benzodiazepinas? *

- Diariamente

- Semanalmente

- Mensualmente

- Menos de una vez al mes

- Solo las he usado una vez

English: How often do you use benzodiazepines?

- Daily

- Weekly

- Monthly

- Less than once a month

- Only once

Q14. ¿Cuál es la razón principal por la que has consumido benzodiazepinas? *

- Para tratar la ansiedad diagnosticada por un médico

- Para controlar el estrés relacionado con los estudios

- Para facilitar el sueño

- Para uso recreativo

- Otra

English: What is the main reason you have used benzodiazepines?

- To treat medically diagnosed anxiety

- To manage study-related stress

- To facilitate sleep

- For recreational use

- Other

Q15. ¿Habías consumido benzodiazepinas antes de comenzar tu carrera? *

- Sí

- No

English: Had you used benzodiazepines before starting your degree?

- Yes

- No

Q16. ¿En qué curso comenzaste a tomar benzodiazepinas? *

- 1º

- 2º

- 3º

- 4º

- 5º

- 6º

English: In which academic year did you start taking benzodiazepines?

- Year 1

- Year 2

- Year 3

- Year 4

- Year 5

- Year 6

Q17. ¿Cómo consideras que te afecta el uso de benzodiazepinas en la atención, concentración y memoria? *

- No noto efectos

- Me hace sentir mejor y me concentro más

- Me cuesta más concentrarme

- No estoy seguro

English: How do you think benzodiazepine use affects your attention, concentration and memory?

- I notice no effects

- I feel better and concentrate more

- It is harder for me to concentrate

- Not sure

Q18. ¿Consideras que dependes de las benzodiazepinas para encontrarte bien? *

- Sí

- No

- No lo sé

English: Do you consider yourself dependent on benzodiazepines to feel well?

- Yes

- No

- I don't know

Q19. ¿Conoces a alguien de tu clase que consuma o haya consumido benzodiazepinas bajo prescripción médica? *

- Sí

- No

- No lo sé

English: Do you know someone in your class who uses/has used benzodiazepines under medical prescription?

- Yes

- No

- I don't know

Q20. ¿Conoces a alguien de tu clase que consuma o haya consumido benzodiazepinas SIN prescripción médica? *

- Sí

- No

- No lo sé

English: Do you know someone in your class who uses/has used benzodiazepines WITHOUT medical prescription?

- Yes

- No

- I don't know

Notes

• The instrument was originally administered in Spanish; the English translation is provided for transparency.

• Items and options are transcribed from the Microsoft Forms™ survey used in the study.

• See manuscript Methods for administration window, data handling, and anonymity settings.
