# Supplementary material for: Use of Benzodiazepines in Medical Students: A Comparative Analysis Between Medical and Other University Degrees
Source: Med Sci (Basel). 2025 Sep 1;13(3):164. doi: 10.3390/medsci13030164 (PMC12452709; doi:10.3390/medsci13030164)
Supplement: Supplementary file 1 [file medsci-13-00164-s001.zip › Supplementary File S2. Original Microsoft Forms.pdf]

CONSUMO DE BENZODIACEPINAS EN ESTUDIANTES DE MEDICINA

ANÁLISIS COMPARATIVO ENTRE ESTUDIANTES DE MEDICINA Y OTROS GRADOS UNIVERSITARIOS

Cuando envíe este formulario, no recopilará automáticamente sus detalles, como el nombre y la dirección de correo electrónico, a menos que lo proporcione usted mismo.

* Obligatorio

CONSENTIMIENTO INFORMADO

Título del Estudio: Consumo de benzodiacepinas en estudiantes de medicina: un análisis comparativo entre estudiantes de medicina y otros grados universitarios

Investigadores Principales: Paula Fernández de Frutos, estudiante de medicina, Universidad Complutense de Madrid; Davide Luordo Tedesco, Profesor asociado a ciencias de la salud, departamento de Medicina, Universidad Complutense de Madrid.

Descripción del Estudio: Le invitamos cordialmente a participar en una encuesta diseñada para investigar el uso de sustancias psicoactivas, con un enfoque particular en las benzodiacepinas, entre estudiantes universitarios.
Las benzodiacepinas son medicamentos psicotrópicos sintéticos, usados en medicina para tratar problemas de ansiedad como el trastorno de ansiedad, el estrés o el insomnio.
Una de las más comunes son diazepam (Valium), alprazolam (Trankimazin) y clonazepam (Rivotril).
Este estudio tiene como objetivo principal analizar la prevalencia de consumo de dichas sustancias entre estudiantes de medicina y compararlo con el de estudiantes pertenecientes a otras disciplinas universitarias. Además, nos interesa determinar cómo los estudiantes acceden a las benzodiacepinas, explorar la relación entre su consumo y el impacto en la capacidad de atención, concentración y memoria, e identificar la prevalencia de uso de benzodiacepinas sin prescripción médica.

Procedimiento: Si acepta participar, se le pedirá que complete una encuesta en línea que indaga sobre su uso de benzodiacepinas y otras sustancias psicotrópicas. Estimamos que completar este cuestionario le tomará entre 10 y 15 minutos.

Voluntariedad y Confidencialidad: Queremos enfatizar que su participación en este estudio es completamente voluntaria. Usted tiene la libertad de retirarse del cuestionario en cualquier momento sin tener que proporcionar una razón y sin ninguna consecuencia. Todas las respuestas que proporcione serán tratadas con la máxima confidencialidad y anonimato. Los datos recogidos serán utilizados únicamente con fines de investigación y no se compartirán con terceros fuera del equipo de investigación.

Beneficios y Riesgos: Su participación nos ayudará a entender mejor el uso de sustancias psicoactivas en el contexto universitario, lo cual puede contribuir a la creación de políticas de salud pública más informadas y efectivas. No se anticipan riesgos significativos por participar en este estudio.

Consentimiento: Al comenzar la encuesta, usted estará dando su consentimiento informado para participar en este estudio bajo los términos aquí descritos. Si tiene alguna pregunta sobre el estudio o sobre sus derechos como participante, por favor, no dude en contactar a los investigadores.

Contacto: Si tiene preguntas o desea más información sobre el estudio, puede contactar a Davide Luordo al davidelu@ucm.es o al 692903919.

Agradecimiento: Apreciamos sinceramente su tiempo y su valiosa contribución a esta investigación. Su participación es esencial para avanzar en nuestro entendimiento sobre este importante tema.

1. HE LEIDO EL CONSENTIMIENTO INFORMADO Y ACEPTO PARTICIPAR EN LA ENCUESTA *

◯ SÍ

◯ NO

No revele nunca su contraseña. Notificar abuso

Microsoft 365

Este contenido lo creó el propietario del formulario. Los datos que envíes se enviarán al propietario del formulario. Microsoft no es responsable de las prácticas de privacidad o seguridad de sus clientes, incluidas las que adopte el propietario de este formulario. Nunca des tu contraseña.

CONSUMO DE BENZODIACEPINAS EN ESTUDIANTES DE MEDICINA

* Obligatorio

DATOS GENERALES

2. EDAD *

- ◯ 18-20 años
- ◯ 21-23 años
- ◯ 24-26 años
- ◯ >26 años

3. SEXO *

- ◯ Hombre
- ◯ Mujer
- ◯ Prefiero no decirlo

4. UNIVERSIDAD DE ORIGEN *

- U. Alcalá de Henares (UAH)
- U. Autónoma de Madrid (UAM)
- U. Carlos III de Madrid (UC3M)
- U. Complutense de Madrid (UCM)
- U. Politécnica de Madrid (UPM)
- U. Rey Juan Carlos (URJC)
- U. Alfonso X el Sabio (UAX)
- U. Antonio Nebrija (Nerbrija)
- U. Camilo José Cela (UCJC)
- U. CEU San Pablo (CEU)
- U. Europea de Madrid (UEM)
- U. Francisco de Vitoria (UFV)
- U. Pontificia de Comillas (UPComillas)
- U. a distancia de Madrid (UDIMA)
- ESIC University
- Otra

5. GRADO AL QUE PERTENECES *

- ADE

- ◯ Derecho
- ◯ Ingeniería Informática
- ◯ Psicología
- ◯ Enfermería
- ◯ Educación primaria
- ◯ Arquitectura
- ◯ Medicina
- ◯ Ingeniería industrial
- ◯ Comunicación audiovisual
- ◯ Fisioterapia
- ◯ Biología
- ◯ Química
- ◯ Educación infantil
- ◯ Ingeniería civil
- ◯ Farmacia
- ◯ Filología hispánica
- ◯ Ciencias ambientales
- ◯ Física
- ◯ Otro

6. DURACIÓN DEL GRADO *

- 4 cursos
- 5 cursos
- 6 cursos
- Opción 4

7. CURSO ACADÉMICO (MARCA EL CURSO EN EL QUE TENGAS MÁS ASIGNATURAS MATRICULADAS) *

- 1º
- 2º
- 3º
- 4º
- 5º
- 6º

No revele nunca su contraseña. Notificar abuso

CONSUMO DE BENZODIACEPINAS EN ESTUDIANTES DE MEDICINA

* Obligatorio

CONSUMO DE BENZODIACEPINAS Y OTRAS SUSTANCIAS PSICOACTIVAS

8. ¿Sabes lo que son las benzodiacepinas? *

- (●) SÍ
- ( ) NO

9. ¿Has tomado alguna vez alguna sustancia psicoactiva (alcohol, drogas, fármacos como las benzodiazepinas) para combatir el estrés de tus estudios? *

- (●) SÍ
- ( ) NO

10. ¿Cuál de las siguientes has usado al menos una vez? *

- ◯ Alcohol
- ◯ Cannabis
- ◯ Cocaína
- ◯ MDMA
- ◯ Anfetaminas ilegales
- ◯ LSD
- ◯ Hongos alucinógenos
- ◯ Ketamina
- ◯ Opiáceos
- ◯ Metanfetamina
- ◯ Metilfenidato
- ◯ Modafinilo
- ◯ Benzodiazepinas
- ◯ Antidepresivos
- ◯ Antipsicóticos
- ◯ ninguna de las anteriores

11. ¿Cómo accediste a las sustancias que has seleccionado sin considerar las que se consideran drogas de abuso? *

- ◯ prescripción médica
- ◯ a través de familiar/amigo
- ◯ Solo he consumido drogas consideradas "de abuso"
- ◯ Otra

12. ¿Has consumido alguna vez benzodiazepinas? *

- ◉ SÍ
- ◯ NO

13. ¿Con qué frecuencia consumes benzodiazepinas? *

- ◯ Diariamente
- ◯ Semanalmente
- ◯ Mensualmente
- ◯ menos de una vez al mes
- ◯ solo las he usado una vez

14. ¿Cuál es la razón principal por la que has consumido benzodiazepinas? *

- ◯ Para tratar la ansiedad diagnosticada por un médico
- ◯ Para controlar el estrés relacionado con los estudios
- ◯ Para facilitar el sueño
- ◯ Para uso recreativo
- ◯ Otra

15. ¿Habías consumido benzodiazepinas antes de comenzar tu carrera? *

- ◯ SÍ
- ◉ NO

16. ¿En qué curso comenzaste a tomar benzodiazepinas? *

- ◯ 1º
- ◯ 2º
- ◯ 3º
- ◯ 4º
- ◯ 5º
- ◯ 6º

17. ¿Cómo consideras que te afecta el uso de benzodiazepinas en la atención, concentración y memoria? *

- No noto efectos
- Me hace sentir mejor y me concentro más
- me cuesta más concentrarme
- no estoy seguro

18. ¿Consideras que dependes de las benzodiazepinas para encontrarte bien? *

- SÍ
- NO
- No lo se

19. ¿Conoces a alguien de tu clase que consuma o haya consumido benzodiazepinas bajo prescripción médica? *

- SÍ
- NO
- No lo se

20. ¿Conoces a alguien de tu clase que consuma o haya consumido benzodiazepinas SIN prescripción médica? *

- SÍ
- NO
- No lo se

No revele nunca su contraseña. Notificar abuso

Microsoft 365

Este contenido lo creó el propietario del formulario. Los datos que envíes se enviarán al propietario del formulario. Microsoft no es responsable de las prácticas de privacidad o seguridad de sus clientes, incluidas las que adopte el propietario de este formulario. Nunca des tu contraseña.

Microsoft Forms | Encuestas, cuestionarios y sondeos con tecnología de inteligencia artificial Crear mi propio formulario

Privacidad y cookies | Términos de uso
